# The Role of Resveratrol in Cancer Therapy

**DOI:** 10.3390/ijms18122589

**Published:** 2017-12-01

**Authors:** Jeong-Hyeon Ko, Gautam Sethi, Jae-Young Um, Muthu K Shanmugam, Frank Arfuso, Alan Prem Kumar, Anupam Bishayee, Kwang Seok Ahn

**Affiliations:** 1 College of Korean Medicine, Kyung Hee University, 24 Kyungheedae-ro, Dongdaemun-gu, Seoul 02447, Korea; gokjh1647@gmail.com (J.-H.K.); jyum@khu.ac.kr (J.-Y.U.); 2 Department for Management of Science and Technology Development, Ton Duc Thang University, Ho Chi Minh City 700000, Vietnam; gautam.sethi@tdt.edu.vn; 3 Faculty of Pharmacy, Ton Duc Thang University, Ho Chi Minh City 700000, Vietnam; gautam.sethi@tdt.edu.vn; 4 Department of Pharmacology, Yong Loo Lin School of Medicine, National University of Singapore, Singapore 117600, Singapore; phcgs@nus.edu.sg (G.S.); phcsmk@nus.edu.sg (M.K.S.); csiapk@nus.edu.sg (A.P.K.); 5 Stem Cell and Cancer Biology Laboratory, School of Biomedical Sciences, Curtin Health Innovation Research Institute, Curtin University, Perth WA 6009, Australia; frank.arfuso@curtin.edu.au; 6 Department of Pharmaceutical Sciences, College of Pharmacy, Larkin University, Miami, FL 33169, USA; abishayee@ularkin.org

**Keywords:** Resveratrol, cancer, molecular targets, apoptosis, chemoprevention, therapy

## Abstract

Natural product compounds have recently attracted significant attention from the scientific community for their potent effects against inflammation-driven diseases, including cancer. A significant amount of research, including preclinical, clinical, and epidemiological studies, has indicated that dietary consumption of polyphenols, found at high levels in cereals, pulses, vegetables, and fruits, may prevent the evolution of an array of diseases, including cancer. Cancer development is a carefully orchestrated progression where normal cells acquires mutations in their genetic makeup, which cause the cells to continuously grow, colonize, and metastasize to other organs such as the liver, lungs, colon, and brain. Compounds that modulate these oncogenic processes can be considered as potential anti-cancer agents that may ultimately make it to clinical application. Resveratrol, a natural stilbene and a non-flavonoid polyphenol, is a phytoestrogen that possesses anti-oxidant, anti-inflammatory, cardioprotective, and anti-cancer properties. It has been reported that resveratrol can reverse multidrug resistance in cancer cells, and, when used in combination with clinically used drugs, it can sensitize cancer cells to standard chemotherapeutic agents. Several novel analogs of resveratrol have been developed with improved anti-cancer activity, bioavailability, and pharmacokinetic profile. The current focus of this review is resveratrol’s in vivo and in vitro effects in a variety of cancers, and intracellular molecular targets modulated by this polyphenol. This is also accompanied by a comprehensive update of the various clinical trials that have demonstrated it to be a promising therapeutic and chemopreventive agent.

## 1. Introduction

Cancer is one of the most commonly diagnosed diseases, and its related morbidity and mortality constitute a very significant health problem worldwide. Although great efforts have been made to discover a cure, cancer remains a very prominent cause of mortality in humans, and effective treatment remains a formidable challenge. An estimated 1.6 million new cancer diagnoses and approximately 600,000 cancer-related deaths are expected in the United States in 2017 alone [1]. Despite several novel improvements in diagnosis and surveillance, the overall cancer survival rate has not improved. Several personalized care medicines, such as targeted therapies, have emerged, providing improved clinical outcomes for cancer patients [2]. However, some of the recent advanced improvements in treating cancer have resulted in development of acquired resistance to chemotherapeutic agents [3]. Carcinogenesis is a multistep and multifactorial process involving the occurrence of clear and discrete molecular and cellular alterations; there are distinct but closely connected phases of initiation, promotion, and progression [4,5,6]. Current cancer therapies, e.g., chemotherapy, targeted agents, radiation, surgery, and immunosuppression, have limitations resulting from the development of resistance to the therapy [7]. The identification of protective molecules without side effects remains a primary objective in the fight against cancer. The other options aim at the early detection of cancer in the benign stage, which can help with its proper management [8].

Since ancient times, natural products have been used to prevent several chronic diseases, including cancer [9,10,11,12,13,14,15,16,17,18]. Revived interest in phytochemicals obtained from dietary or medicinal plant sources has provided an alternative source of bioactive compounds that can be used as preventive or therapeutic agents against a variety of diseases [19,20,21,22,23]. Phytochemicals such as phytoestrogens have been reported to modulate multiple cellular-signaling pathways, with no or minimal toxicity to normal cells [24,25]. The application of substances to prevent or delay the development of carcinogenesis has been termed chemoprevention [4], and there is burgeoning interest in the use of natural compounds as possible chemopreventive and therapeutic agents for human populations. Resveratrol is increasing in prominence because it has cancer-preventive and anti-cancer properties [25,26,27,28]. A non-flavonoid polyphenol, resveratrol (3,4′,5-trihydroxy-*trans*-stilbene) is a phytoalexin that naturally occurs in many species of plants, including peanuts, grapes, pines, and berries, and assists in the response against pathogen infections [29]. Interestingly, Chinese and Japanese traditional medicine also contain it, in the form of extracts such as those obtained from *Polygonum cuspidatum*, which can be used to treat inflammation, headaches, cancers, and amenorrhea.

**Figure 1:**
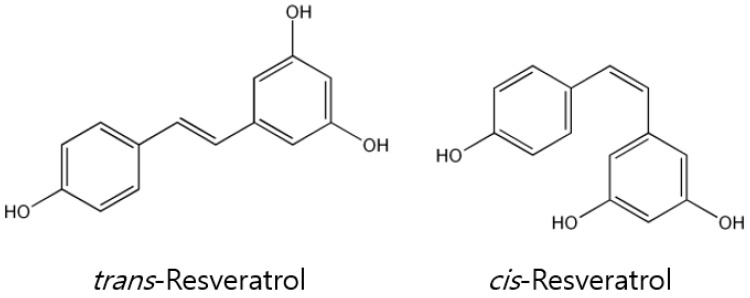
The chemical structure of two geometric isomers of resveratrol.

The structure of resveratrol is stilbene-based and comprises two phenolic rings connected by a styrene double bond to produce 3,4′,5-trihydroxystilbene, which occurs in both the *trans*- and *cis*-isoforms (Figure 1). The *trans*-isoform is the major isoform, and represents the most extensively studied chemical form. Exposure to heat and ultraviolet radiation can cause the *trans*-isoform to convert into the *cis*-isoform, whose structure closely resembles that of the synthetic estrogen diethylstilbestrol. Because of this, resveratrol has also been classified as a phytoestrogen. Its biosynthetic pathway begins with a reaction between the malonyl CoA and coumaryl derivative, which is catalyzed by the enzyme stilbene synthase [30]. Resveratrol is easily available in a regular diet and has numerous health-augmenting properties, as well as some naturally occurring analogs, such as viniferins, pterostilbene, and piceid [31]. Additionally, some semi-synthetic resveratrol analogs have also been found to have certain pharmacological benefits, including chemoprevention actions, anti-oxidant effects, and anti-aging properties [32,33,34]. It had also been shown that resveratrol can reverse drug resistance in a variety of tumor cells by sensitizing them to chemotherapeutic agents [35,36]. In particular, it has been reported that *trans*-resveratrol and its glucoside have wide-ranging effects, including cardioprotective, anti-oxidative, anti-inflammatory, estrogenic/anti-estrogenic, and anti-tumor properties [37,38]. Moreover, the antimicrobial effects [39] of *trans*-resveratrol have been found to be useful in the management of cognitive disorders such as dementia [40,41]. This review, however, will concentrate primarily on resveratrol and discuss its diverse anti-cancer effects in various preclinical and clinical studies.

## 2. In Vitro Pharmacological Properties and Anti-Cancer Effects of Resveratrol

It has been shown that resveratrol possesses multifaceted salubrious properties, e.g., anti-inflammatory, anti-oxidative, and anti-aging qualities [42,43,44]. Resveratrol is a constituent of red wine, and therefore it is often postulated that resveratrol is a significant element in the French Paradox, the reduced risk of cardiovascular disease in French populations despite the high intake of saturated fats; that has been associated with high red wine consumption [45]. After Jang et al. [46] found that resveratrol inhibited carcinogenesis in a mouse-skin cancer model in 1997, a wealth of publications followed. It has been shown that resveratrol has in vitro cytotoxic effects against a large range of human tumor cells, including myeloid and lymphoid cancer cells, and breast, skin, cervix, ovary, stomach, prostate, colon, liver, pancreas, and thyroid carcinoma cells [25,47,48,49]. Resveratrol affects a variety of cancer stages from initiation and promotion to progression by affecting the diverse signal-transduction pathways that control cell growth and division, inflammation, apoptosis, metastasis, and angiogenesis.

## 3. Anti-Tumor-Initiation Activity

Neoplasia initiation concerns the alteration or mutation of genes resulting spontaneously from or caused by exposure to a carcinogenic agent, which finally results in mutagenesis [50]. Oxidative stress plays a dominant part in the causation of carcinogenesis [51]. Reactive oxygen species (ROS) can react with DNA in addition to chromatin proteins, resulting in several types of DNA damage [52,53]. In fact, chemical carcinogens cannot damage DNA until they are metabolized by phase-I biotransformation enzymes, especially cytochrome P450, in cells and converted to reactive electrophiles. In addition, carcinogen-DNA adduct formation gives rise to chemical carcinogenesis [54]. This initiation stage is irreversible but can be prevented by inhibiting the activity and expression of certain cytochrome P450 enzymes and augmenting the activity of phase-II detoxification enzymes, which transform carcinogens into less toxic and soluble products [55,56].

It has been found that resveratrol can inhibit events linked to the initiation of tumors. For instance, resveratrol treatment suppressed free radical formation induced by 12-*O*-tetradecanoylphorbol-13-acetate (TPA) in human leukemia HL-60 cells [57]. The diverse anti-oxidant properties of resveratrol have already been described previously [58,59]. Resveratrol is an excellent scavenger of hydroxyls and superoxides, as well as radicals induced by metals/enzymes and generated by cells [59]. It also protects against lipid peroxidation within cell membranes and damage to DNA resulting from ROS [59]. Furthermore, resveratrol functions as an anti-mutagen, as shown by its inhibition of the mutagenicity of N-methyl-N’-nitro-N-nitrosoguanidine in the *Salmonella typhimurium* strain TA100 [60]. It has been proposed that resveratrol can be a possible chemopreventive agent, and its anti-mutagenic and anti-carcinogenic properties have been demonstrated in several models [9,61,62].

In addition, resveratrol can inhibit 2,3,7,8-tetrachlorodibenzo-p-dioxin (TCDD)–induced expression of cytochrome P450 1A1 (CYP1A1) and 1B1 (CYP1B1), as well as their catalytic actions, in human breast epithelial Michigan cancer foundation (MCF)-10A cells [63]. Resveratrol can also abrogate the CYP1A activity induced by environmental aryl hydrocarbon benzo[a]pyrene (B[a]P) and catalyzed by directly suppressing the CYP1A1/1A2 enzyme activity and the signal-transduction pathway that up-regulates the expression of carcinogen-activating enzymes in human breast cancer MCF-7 and liver cancer HepG2 cells [64]. It has been reported that resveratrol also has inhibitory effects on aryl hydrocarbon receptor (AhR)–mediated activation of phase-I enzymes. The canonical AhR-dependent signaling pathway is thought to contribute to carcinogenic initiation by phase-I enzyme–activated polycyclic aromatic hydrocarbons (PAH). Briefly, PAH can bind to the AhR and facilitate its translocation into the nucleus, where the AhR develops into a heterodimer with AhR nuclear translocator (ARNT). The AhR/ARNT heterodimer then attaches to and transactivates xenobiotic response element–driven phase-I/II enzyme promoters, and initiates carcinogenesis. It has been postulated that resveratrol’s inhibition of AhR signaling can suppresses this initiation process. For example, resveratrol caused inhibition of TCDD-induced recruitment of AhR and ARNT to the CYP1A1/1A2 and CYP1A1/1B1 promoter in HepG2 and MCF-7 cells, respectively, culminating in decreased expression [65]. Resveratrol also reduced TCDD-induced, AhR-mediated CYP1A1 expression in gastric cancer AGS cells [66]. Resveratrol could therefore modulate the activity and expression of some cytochrome P450 enzymes, and thereby help prevent cancer by limiting the activation of pro-carcinogens.

It has also been found that resveratrol increases both the activity and expression of NAD(P)H: quinone oxidoreductase-1 (NQO1), a carcinogen-detoxifying phase-II enzyme, in human leukemia K562 cells [67]. In addition, resveratrol was also found to induce the activity of the phase-II detoxifying metabolic enzyme quinone reductase (QR) within mouse liver-cancer Hepa 1c1c7 cells [68]. Within breast cancer cells, resveratrol induced QR expression via the estrogen receptor β (ER-β), thereby protecting against oxidative damage to DNA [69]. Resveratrol also augments the activity and expression of anti-oxidant and phase-II detoxifying enzymes through the activation of nuclear factor E2–related factor 2 (Nrf2). Nrf2 generally remains sequestered in the cytoplasm by binding Kelch-like ECH-associated protein 1 (Keap1). When Nrf2 is induced by dietary phytochemicals like resveratrol, it dissociates itself from Keap1 and translocates into the nucleus. Nrf2 thereafter attaches to the anti-oxidant response element (ARE) found in the promoters of several genes that encode phase-II enzymes, and thus regulates their transcriptional activation [70,71]. Resveratrol has been also shown to up-regulate the expression of heme oxygenase-1 (HO-1) via Nrf2 activation in PC12 cells. Resveratrol induction of the expression of NQO1 in TCDD-treated normal human breast epithelial MCF10F cells involved Nrf2, resulting in the formation of DNA adducts being suppressed [72].

Resveratrol also caused an increase in NQO1 after estradiol-3,4-quinone (E_2_-3,4-Q) or 4-hydroxyestradiol (4-OHE_2_) treatment in MCF10F cells [73]. In addition, resveratrol-induced Nrf2 signaling can lead to an increased expression of HO-1, NQO1, and the glutamate cysteine ligase (GCL) catalytic subunit in human bronchial epithelial HBE1 cells treated with cigarette-smoke extracts [74]. Resveratrol also restored glutathione levels in human lung cancer A549 cells treated with cigarette-smoke extracts, by Nrf2-induced GCL expression [75]. In leukemia K562 cells resveratrol increased NQO1 expression and induced Nrf2/Keap1/ARE binding to NQO1 promoter [67].

## 4. Anti-Tumor-Promotion Activity

Tumor promotion involves clonally enlarging initiated cells to create a continuously proliferating, premalignant lesion. Tumor promoters generally alter gene expression, resulting in increased cell proliferation and decreased death of cells [76]. Studies conducted in vitro have discovered that resveratrol exerts an anti-proliferative activity by inducing apoptosis. Of these, resveratrol modifies the balance of cyclins as well as cyclin-dependent kinases (CDKs), resulting in cell cycle inhibition at G0/G1 phase. For example, a link has been found between the inhibition of cyclin D1/CDK4 by resveratrol and cell cycle arrest in the G0/G1 phase within different cancer cells [77,78,79,80]. Resveratrol was also shown to increase the levels of cyclin A and E, with cell cycle arrest in the G2/M and S phases [81,82]. Similar findings have indicated that resveratrol causes the arrest of cell cycles and activation of the p53-dependent pathway [83,84,85].

p53, a tumor-suppressor protein, is an element critically linked to transcription, and is closely connected to the regulation of apoptosis and cell proliferation; and also acts as a key mediator in the prevention of carcinogenesis [86]. p53 that has been activated binds DNA and stimulates the expression of certain genes, e.g., *WAF1*/*CIP*1 encoding for p21, which belongs to the group of CDK inhibitors that are vital to the inhibition of cell growth [87]. Resveratrol reduced the development of human skin cancer A431 cells by downregulating the expression of cyclin D1, cyclin D2, and cyclin E, inhibiting the activities and/or expression of CDK2, CDK4, and CDK6, and upregulating the expression of p21. Resveratrol also suppressed the proliferation of breast cancer MCF-7 and human prostate cancer DU-145 cells [88] via modulating CDK4 and cyclin D1 expression, which have been linked to the induction of p21 and p53. When used to treat A549 cells, resveratrol caused S phase arrest, reduced retinoblastoma protein (Rb) phosphorylation, and induced p21 and p53 protein expression [89]. It has also been demonstrated that resveratrol limits the expression of Rb, another tumor-suppressor protein involved in the G1/S transition in normal conditions [79,82,85].

It has also been shown that resveratrol’s anti-proliferative activity involves the stimulation of apoptosis within cancer cells [90,91,92]; it has been proposed that apoptosis activation could be a probable mechanism for chemotherapeutic agents to destroy cancerous cells [93,94]. In many human tumors, apoptosis has been found to be impaired, which suggests that the disruption of apoptotic functions significantly contributes to a normal cell being transformed into a tumor cell. Apoptosis is cell death that has been programmed, and a genetically regulated physiological mechanism to eliminate damaged or abnormal cells. It is also significant as a physiological-growth-control regulator and a tissue-homeostasis moderator in embryonic, fetal, and adult tissues. Apoptotic cells can be identified by regular biochemical and morphological properties, including membrane blebbing, cell shrinkage, nuclear DNA fragmentation, chromatin condensation, and formation of apoptotic bodies [95].

Apoptosis can be activated via two major pathways: the mitochondria-apoptosome-mediated intrinsic pathway and the death receptor–induced extrinsic pathway. [96,97]. The triggering of death receptors in the tumor necrosis factor (TNF) receptor superfamily, e.g., Fas (CD95/APO-1), or of TNF-related apoptosis-inducing ligand (TRAIL) receptors causes the initiator caspase-8 to be activated, which can mediate the apoptosis signal via direct cleavage of downstream effector caspases such as caspase-3 [98]. Caspases are an ubiquitous family of cysteine proteases, and have critical functions in apoptosis as upstream initiators and downstream effectors [99]. The intrinsic pathway is triggered by the dispensation of apoptogenic factors such as Omi/HtrA2, Smac/DIABLO, cytochrome c, apoptosis-inducing factors (AIFs), endonuclease G, caspase-2, or caspase-9 from the mitochondrial intermembrane space [100]. The dissemination of cytochrome c into the cytosol activates caspase-3 via the creation of the cytochrome c/apoptotic protease-activating factor-1 (Apaf-1)/caspase-9-containing apoptosome complex; Omi/HtrA2 and Smac/DIABLO encourage caspase activation by neutralizing the effects of inhibitors of apoptotic proteins (IAPs) [100,101].

Crosstalk also occurs between the two apoptotic pathways. For instance, Fas is connected to the intrinsic pathway that is regulated via the activation of caspase-8 to cause cleavage of the BID protein, causing cytochrome *c* to be released from the mitochondria [102,103]. Various apoptotic cell-death mechanisms have been propounded [104,105]. One logical approach to reducing the incidence of cancer appears to be the targeting of critical parts of apoptosis regulatory pathways, including the IAPs (in particular XIAP, cIAP1, and cIAP2), the anti-apoptotic Bcl-2 family of proteins, nuclear factor-kappa B (NF-κB), survivin, tyrosine kinases, caspases, and critical signaling pathways (phosphoinositide 3-kinase (PI3K)/AKT, STAT3/5, and MAPK pathways) [7,13,20,106,107,108,109,110,111,112]. Resveratrol prompts the death of tumor cells by modulating diverse signal transduction pathways via regulation of the levels of Fas and Fas-ligand (FasL) [113,114]. Resveratrol also enhances FasL expression in HL-60 cells, and the resveratrol-induced apoptosis is Fas signaling-dependent [113].

Similar outcomes have also been observed in breast [113] and colon cancer cells [114]. Mechanisms of cell death that are independent of Fas and caused by cytotoxic agents have also been propounded [115,116], and apoptosis induced by doxorubicin occurs through a Fas-independent pathway [116]. Likewise, it has been shown that resveratrol exhibits Fas-independent apoptosis in another leukemic THP-1 cell line [117]. It has also been observed that resveratrol induced the death of leukemia CEM-C7H2 cells in a Fas-independent manner, as demonstrated by the absence of apoptotic change in the presence of antibodies antagonistic to Fas or FasL [118]. Furthermore, resveratrol effectively triggered apoptosis in Fas-resistant Jurkat human leukemia cells [118].

It has been shown that resveratrol induces cell death in some cancer cells by changing the proteins of the Bcl-2 family [119]. The inhibition of anti-apoptotic proteins of the Bcl-2 family, and activation of pro-apoptotic proteins such as Bad, Bak or Bax, by resveratrol has also been shown to be a mechanism for caspase activation and cytochrome *c* release [120,121]. Interestingly, these effects may be correlated with p53 activation [122,123,124,125]. For instance, resveratrol increased the cytoplasmic concentration of calcium in human breast cancer MDA-MB-231 cells, which activated p53 and caused different pro-apoptotic genes to be transcribed [126].

It has also been shown that resveratrol induces apoptosis via inhibiting the PI3K/Akt/mTOR pathway [79,120,127,128,129,130,131], modulating the mitogen-activated protein kinase pathway (MAPK) [129,130,132], and inhibiting NF-κB activation [133,134]. Resveratrol triggered apoptosis within human T-cell acute lymphoblastic leukemia MOLT-4 cells by abrogating Akt phosphorylation, and subsequently preventing GSK3β from being activated [135]. Similarly, resveratrol induced apoptosis in ovarian, [136] breast, [137] uterine, [138] prostate, [120] and multiple myeloma cells [121], via inhibiting Akt phosphorylation. Chen et al. [139] determined that resveratrol inhibited the phosphorylation of PI3K/Akt (i.e., PI3K/Akt inactivation) in prostate cancer cells, resulting in decreased Forkhead box protein (FOXO) activation. Resveratrol’s inhibition of the serine/threonine protein kinase Akt has been identified in anti-cancer activity modulated by the activation of FOXO3a in human breast cancer cells, because FOXO3a was not found to be activated by Akt [140].

It has been suggested that resveratrol interferes with the MAPK pathway. In cervical carcinoma cells, resveratrol inhibited the activation of p38, JNK1, and ERK2 [141]. Resveratrol activates ERK1/2 at low concentrations (1 pM–10 μM), but at higher concentrations (50–100 μM) can inhibit MAPK in human neuroblastoma SH-SY5Y cells [142]. In contrast, resveratrol activates ERK1/2 in prostate [143], breast [144,145], glial [146], head and neck [147], and ovarian cancer cells [148]. MAPKs in a constitutively active state are necessary to maintain the malignant state; however, short-term activation of MAPK may drive the cells to apoptosis [149]. It has also been reported that resveratrol causes activation of other kinases, like JNK and p38 [150]. Notably, it has been shown that the resveratrol’s anti-tumor effects require p53 activation that is MAPK-induced, as well as the subsequent induction of apoptosis [151,152,153].

Resveratrol induces apoptosis in, and obstructs proliferation of, human multiple myeloma cells via inhibiting the constitutive activation of NF-κB through abrogating the IκB-α kinase activation, and thus down-regulating certain anti-apoptotic and pro-proliferation gene products, such as survivin, cIAP-2, cyclin D1, XIAP, Bcl-xL, Bfl-1/A1, Bcl-2, and TNF-α receptor-associated factor 2 (TRAF2) [121,154]. The constitutive activation of NF-κB, defined as the persistence of NF-κB within the nucleus, is apparent in a wide range of cancer cells [155,156,157,158]. Active NF-κB drives the expression of a plethora of genes that guard against apoptotic cell death and maintain cell proliferation [158]. Deregulation of the NF-κB signaling pathway can cause increased apoptosis as NF-κB modulates anti-apoptotic genes, e.g., TRAF1 and TRAF2, and thus changes the activities of caspases critical to the majority of apoptotic processes [159]. It has been determined that resveratrol can suppress NF-κB-regulated gene products connected with inflammation matrix metalloproteinase (MMP)-3, MMP-9, cyclooxygenase-2 (COX-2), and vascular endothelial growth factor (VEGF), inhibit anti-apoptotic proteins (Bcl-xL, Bcl-2, and TRAF1), and activate cleaved-caspase-3 [160].

Resveratrol also causes inhibition of signal transducers and activators of transcription 3 (STAT3), which adds to its pro-apoptotic and anti-proliferative potential [121]. STAT3 is a critical element in inflammation-related tumorigenesis as it promotes the proliferation, survival, invasion, angiogenesis, and metastasis of tumor cells [112,161]. The activation of NF-κB also promotes inflammation, proliferation, and tumorigenesis [162]. STAT3 and NF-κB are two central transcriptional factors linking tumorigenesis and inflammation; both of them can be activated as a response to certain stimuli, such as cytokines, growth factors, and stress signals. Abnormal signaling of STAT3 or NF-κB in malignant cells is therefore a promising target of therapy. STAT3 and NF-κB are activated via distinct pathways, and move to the nucleus to effect transcriptional activity. STAT3 and NF-κB that are constitutively activated by acetylation and/or phosphorylation in tumor cells, have been closely linked to both cancer development and progression [163,164]. Kim et al. reported that resveratrol caused inhibition of the nuclear translocation of STAT3 in renal cell carcinoma [165].

Interestingly, Wen et al. showed that inhibiting NF-κB nuclear translocation caused apoptosis in resveratrol-treated medulloblastoma cells [166]. It has been suggested that cross-talk occurs between the STAT3 and NF-κB pathways, because of the release of IL-6 and other cytokines, and because of the activation of cytokine receptors. STAT3 and NF-κB actually co-regulate many inflammatory and oncogenic genes, like *IL-1β*, *Bcl-xL*, *Myc*, *COX-2*, and *cyclin D1* [161]. By their possible functional interaction, STAT3 and NF-κB collaboratively promote the development of tumors via inducing the expression of pro-tumorigenic genes [167]. The dysregulation of these genes because of the constant activation of both STAT3 and NF-κB in tumors and the tumor microenvironment is critical to tumor progression. Inflammation can regulate angiogenesis and cellular proliferation, and inhibits apoptosis [168]. It has also been reported that resveratrol inhibits the processes of several inflammatory enzymes in vitro, e.g., COXs and lipoxygenases (LOXs) [169,170]. It was shown in a recent study that resveratrol could radiosensitize and block the STAT3 signaling pathway by inducing SOCS-1, thereby reducing STAT3 phosphorylation and proliferation in head and neck tumor cells [171].

## 5. Anti-Tumor-Progression Activity

Tumor progression involves several processes such as that lead to tumor metastasis. Several genes are mutated or deleted that sustain the development of aggressive tumors. The invasion and metastasis of cancer cells involve the destruction of the extracellular matrix (ECM) and basement membrane, by proteolytic enzymes, such as matrix metalloproteinases (MMPs). Of these enzymes, MMP-2 and MMP-9 are overexpressed within a variety of malignant tumors modulating cell invasion and metastasis [172]. Tissue inhibitor metalloproteinase proteins (TIMPs), on the other hand, are a protein group comprising TIMP-1, -2, -3, and -4 acting as natural MMP inhibitors [173]. To sustain their swift growth, invasive tumors also need to grow new blood vessels via a process called angiogenesis. During angiogenesis, endothelial cells can be stimulated by various growth factors, including fibroblast growth factor (FGF) and VEGF, and travel to where the new blood vessels are required. Blocking the development of new blood vessels causes the supply of nutrients and oxygen to be reduced and, as a result, the size of the tumor and metastasis may also be reduced.

It has been suggested that resveratrol plays a role in inhibiting the expression of MMP (mainly MMP-9) [174,175,176,177] and angiogenesis markers such as VEGF, EGFR or FGF-2 [79,178]. Resveratrol reduced the phorbo-12-myristate 13-acetate (PMA)-induced migration and invasion ability of liver cancer HepG2 and Hep3B cells. In HepG2 cells, resveratrol up-regulated TIMP-1 protein expression and down-regulated MMP-9 activity, while the activities of MMP-2 and MMP-9 were decreased, along with a rise in the protein-expression level of TIMP-2 in Hep3B cells [175]. HepG2 cells treated with TNF-α expressed a high level of MMP-9, which resveratrol suppressed considerably via down-regulating the expression of NF-κB, resulting in the expression of MMP-9 protein being suppressed and the invasive capability of HepG2 cells being diminished [174]. Resveratrol treatment of breast cancer MDA-MB231 cells caused inhibition of the epidermal growth factor (EGF)-induced elevation of cell migration, and of the expression of MMP-9. Resveratrol also reduced a subunit of the mammalian mediator complex for transcription (called MED28, and whose over-expression can increase migration), via the EGFR/PI3K signaling pathways [176]. Both VEGF and hypoxia-inducible factor-1α (HIF-1α) are over-expressed in several human tumors and their metastases, and are closely linked to a more aggressive tumor phenotype. It has been reported that resveratrol suppresses the expression of VEGF and HIF-1α in human ovarian cancer cells via abrogating the activation of the PI3K/Akt and MAPK signaling pathways [179]. Resveratrol caused inhibition of the expression of these molecules, which suggests that it could be part of an efficacious anti-cancer therapy for preventing cancer and its metastasis [180,181,182].

Malignant transformation may be linked to signaling pathways during tumorigenesis, thereby promoting epithelial-to-mesenchymal transition (EMT), which may in turn increase the invasiveness and motility of cancer cells, and trigger cancer metastasis [183,184]. Many studies have shown that resveratrol suppresses the development of tumor invasion and metastasis through inhibiting signaling pathways associated with EMT [185]. Transforming growth factor-beta (TGF-β) is a widely known cytokine that encourages invasion, proliferation, EMT, and angiogenesis of cancer cells, and the TGF-β/Smad signaling pathway can activate EMT during cancer metastasis [186,187]. Resveratrol (20 μM) inhibited TGF-β-induced EMT in A549 lung cancer cells by augmenting the expression of E-cadherin and attenuating the expression of vimentin and fibronectin, as well as the EMT-inducing transcription factors Slug and Snail [188]. Qing Ji et al. showed that resveratrol inhibited EMT induced by TGF-β, as well as the invasion and metastasis of colorectal cancer, via reducing Smad2/3 expression [189]. NF-κB can also promote EMT, in addition to cancer migration and invasion [190,191,192].

Several studies have shown that NF-κB is a significant EMT regulator for different types of cells [190,191,192,193,194]. The roles for NF-κB have been found to be linked to the expression of various genes related to EMT, such as *ZEB1*, *Sna*il, *E-cadherin*, *MMP-7*, *MMP-9*, and *MMP-13* [192,193,195,196]. NF-κB can also be activated through PI3K/Akt signaling pathway to drive EMT and cancer-cell metastasis. Resveratrol suppressed the metastatic potential of pancreatic cancer PANC-1 cells in vitro by regulating factors related to EMT (vimentin, E-cadherin, N-cadherin, MMP-2, and MMP-9) and modulating the activation of PI3K/Akt/NF-κB pathways [197].

## 6. Pre-Clinical Studies

Resveratrol has also been reported to possess a significant anti-cancer property in various preclinical animal models (Table 1).

## 7. Skin Cancer

The first preclinical study of the anti-cancer or chemopreventive effect of resveratrol was reported in a two-stage, 7,12-Dimethylbenz[a]anthracene (DMBA)-initiated and 12-*O*-tetradecanoyl-13-acetate (TPA)-promoted mouse-skin carcinogenesis model [46]. Thereafter, several in vivo skin cancer studies have been performed with DMBA/TPA [46,199,200,201,235,236], DMBA alone, [237,238,239], TPA alone [240,241,242], ultraviolet B radiation (UVB) exposure [202,203,204,243], benzo[a]pyrene (BP) [237], and xenograft models [198]. In the DMBA/TPA models, resveratrol treatment reduced the incidence [46,199,200,201,235], multiplicity [46,199,201,235], and tumor volume [201,235,236], and delayed the onset of tumorigenesis [201]. Resveratrol prevented DMBA/TPA-induced skin cancer from developing in mice, and was effective at all stages of carcinogenesis.

Soleas et al. discovered that resveratrol was somewhat efficacious in reducing the rate of tumor formation and the number of animals that developed skin tumors induced by DMBA [200]. Resveratrol inhibited tumor promotion in the DMBA–TPA mouse-skin carcinogenesis model, possibly because (at least in part) of its anti-oxidant properties [199]. Resveratrol administration restored glutathione (GSH) levels, superoxide dismutase (SOD), GSH peroxidase, and catalase activities to control values (mice without UVB irradiation) [244]. Furthermore, resveratrol exerted an anti-oxidant effect with a reduction in H_2_O_2_ and lipid peroxidation in the skin [202]. It has been shown that the anti-proliferative effects of this stilbene can be regulated by cell-cycle regulatory proteins such as the expression of CDK2, 4, and 6, cyclin D1 and D2, and proliferating cell nuclear antigen (PCNA), while the expression of p21 was increased [203].

Resveratrol effectively hindered the development of DMBA/TPA-induced mouse-skin tumors by inducing apoptosis, which was indicated by the induction of cytochrome *c* release, the expression of Bax, p53, and Apaf-1, and the inhibition of Bcl-2 [201]. Afaq et al. determined that resveratrol had the ability to reduce edema and inflammation resulting from short-term UVB exposure in the skin of SKH-1 hairless mice, possibly because of the inhibition of ornithine decarboxylase (ODC) [202]. Treatment with resveratrol both before and after exposure to UVB suppressed development of skin tumor [204]. Resveratrol’s anti-tumor properties have also been linked to lower expression levels of TGF-β1 and augmented expression levels of E-cadherin [243]. Oral gavage of resveratrol hindered the development of a mouse melanoma (B16BL6 cell line) xenograft carried in mice, with decreased expression of Akt [245]. In a murine model of the human cutaneous skin squamous carcinoma A431 cell-line xenograft, resveratrol treatment reduced the volume of the tumor, raised the expression levels of ERK and p53, and lowered the expression level of survivin [198]. Nevertheless, resveratrol did not reduce the tumor growth of other melanoma cell lines, including A375, B16M, and DM738 xenografts in mice [246,247].

## 8. Breast Cancer

Resveratrol has exhibited anti-cancer and chemopreventive properties in various animal breast cancer models. Models of chemically induced mammary-gland carcinogenesis using N-methyl-nitrosourea (MNU) [212], estradiol [248], or DMBA [46], in addition to models of spontaneous mammary tumors with HER-2/neu-overexpressed [207] or Brca1-mutated (K14cre; Brca1F/F; p53F/F) mice [249], have been employed to determine resveratrol’s preventive or curative effects. Oral administration of resveratrol was also found to reduce tumorigenesis induced by N-nitoso-N-methylurea (NMU) in rats [212,250].

Resveratrol, in a xenograft animal model, inhibited the development of ER-β–positive MDA-MB-231 and estrogen receptor (ER)-α–negative tumor explants, raised apoptosis, and lowered angiogenesis in nude mice [208]. However, resveratrol did not affect the in vivo development and metastasis of transplanted ER-α–negative 4T1 murine mammary cancer cells in nude mice [251]. Bove et al. studied resveratrol’s in vivo effect with doses of 1–5 mg/kg per day administered intraperitoneally, and proposed that this ineffectiveness may have been the result of an insufficient dose of resveratrol. In another study, oral resveratrol at 100 or 200 mg/kg inhibited the development of 4T1 cells and metastasis in mouse lungs [252]. These findings were linked to both the reduced activity and expression of MMP-9. These data suggest that resveratrol’s effects on breast cancer hinge on the dose and route of administration.

With breast cancer cell–implanted fat-pad models employing cigarette smoke condensate–transformed MCF-10ATr cells [209] or SUM159 cells [253], resveratrol caused down-regulation of the expression of various proteins linked to survival and cell proliferation (cyclin D1, PI3K, PCNA, and β-catenin), proteins related to DNA repair (Fen-1, DNA-ligase-I, Pol-δ, and Pol-ε), and an anti-apoptotic protein (Bcl-xL). It also caused an up-regulation of the pro-apoptotic protein Bax and tumor-suppressor gene p21 in mouse mammary tissue [209,253]. When used to supplement drinking water, resveratrol delayed the growth of spontaneous mammary tumors in HER-2/neu transgenic mice, and lowered the mean size and number of mammary tumors by causing down-regulation of the HER-2/neu gene expression and raising apoptosis in the mammary glands of these mice [207].

## 9. Prostate Cancer

Dietary resveratrol considerably lowered the incidence of prostatic adenocarcinoma in the transgenic adenocarcinoma mouse prostate (TRAMP) model [216]. Resveratrol suppressed prostate cancer growth via down-regulating the androgen receptor (AR) expression in the TRAMP model of prostate cancer. Additionally, besides down-regulating the AR expression, resveratrol also suppressed the mRNA level of androgen-responsive glandular kallikrein 11, which has been determined to be an ortholog of the human prostate specific antigen (PSA) [217]. In a xenograft model, resveratrol delayed the development of AR-positive LNCaP tumors and inhibited the expression of steroid hormone response markers [254].

With the use of AR-negative PC-3 human prostate cancer–cell xenografts in the flank regions of mice, post-treatment with oral resveratrol (30 mg/kg/day) decreased the volume of tumors, with lowered tumor-cell proliferation and neovascularization, and induced apoptosis [214]. Intraperitoneal post-treatment with resveratrol (25 mg/kg/day) also decreased the tumor volume of PC-3 cell xenografts in mouse prostates [255]. Additionally, intraperitoneal post-treatment of resveratrol (50 mg/kg/day) in the orthotopic DU-145 prostate cancer model decreased the growth, progression, local invasion, and spontaneous metastasis of tumors [215].

## 10. Colorectal Cancer

Colorectal cancers arise due to several factors such as diet rich in red meat and processed meat and other lifestyle factors such as smoking and drinking alcohol [256],. Resveratrol’s in vivo effectiveness has been tested with colorectal cancer models employing genetically modified animals such as Apc*^Pirc^*^/*+*^ rats and Apc*^Min^*^/*+*^ mice. Colon cancer can be induced by chemical carcinogens, which include azoxymethane (AOM), AOM plus dextran sulfate sodium (DSS), 2-amino-1-methyl-6-phenylimidazo[4,5-b]pyridine, 2-amino-3-methylimidazo[4,5-f]quinoline, and 1,2-dimethylhydrazine (DMH) [257,258]. The pathophysiological and histopathological features/manifestations of colon cancer include aberrant crypt foci (ACF), hyperplasia, adenocarcinoma, and adenoma [258]. In models induced with AOM or AOM plus DSS, the oral administration (in the gavage or diet) of resveratrol decreased the incidence [259,260], individual size [224], and multiplicity [224,259,261] of ACF in rodent models, and triggered biomarker alterations.

Resveratrol augmented the expression of Bax [224], p53, and p-p53 at Ser15 [259], HO-1 [261], glutathione reductase (GR) [261], and Nrf2 [261], and reduced the expression of COX-2 [259,261], inducible nitric oxide synthase (iNOS) [259,261], TNF-α [259], aldose reductase [261], NF-κB [261], and p-protein kinase C-β2 (PKC-β2) [261]. It has been propounded that resveratrol down-regulates the aldose reductase–dependent activation of NF-κB and PKC-β2, with an ensuing lowering of the expression levels of COX-2 and iNOS [261]. In models induced with DMH, resveratrol decreased the incidence, [222] size [222,262], and multiplicity of ACF [222,262,263], as well as histopathological lesions [222] and DNA damage in leukocytes [264]. When used against colon carcinogenesis, the anti-tumor effects of resveratrol were found to be accompanied by alterations in the activities of enzymes. In rat models, the processes of anti-oxidant enzymes, including catalase (CAT) and SOD in the intestine/colon [262], liver [265], and erythrocytes [264], were augmented, and the processes of biotransforming enzymes, including β-glucosidase, β-glucuronidase, β-galactosidase, nitroreductase, and mucinase, in fresh fecal and colonic mucosal samples were reduced [222]. Resveratrol lowered the expression levels of ODC, COX-2, Mucin 1, cell surface associated (MUC1), heat-shock protein (Hsp)27, and Hsp70 in colonic mucosa [266], and increased the expression levels of caspase-3 in the colonic mucosa [266], and increased glutathione in the reduced state (GSH) in the liver, intestine/colon, plasma, and erythrocytes [262,264,265].

In models with genetically modified mice (e.g., Apc*^Min^*^/*+*^ mice [223,225,226]), and in mice with the APC locus knockout and activated *KRAS* [267], resveratrol supplementation inhibited the development of colon tumors [223,225,226,267,268] and occurrence of dysplasia [223].

## 11. Liver Cancer

The anti-cancer potential of resveratrol in liver carcinogenesis was exemplified by a decreased incidence and smaller numbers of nodules in models of animals employing chemical carcinogens [e.g., diethylnitrosamine (DENA) [269], DENA plus phenobarbital [234,270], and DENA plus 2-acetylaminofluorene (2-AAF) [271] or transgenic mice (e.g., hepatitis B virus X protein (HBx)-expressing transgenic mice) [272]. Additionally, resveratrol’s anti-tumor effects have been reported in xenograft models using hepatoma cell lines (e.g., H22, AH-130, HepG2, and AH109A) [227,228,229,232]. Dietary resveratrol completely prevented DENA-induced lipid peroxidation and enhanced protein carbonyl formation, which indicates that it may also attenuate oxidative stress in the liver. Resveratrol also elevated the expression of hepatic Nrf2 and reduced the expression of iNOS. That study reported that the attenuation of oxidative and nitrosative stress and the alleviation of the inflammatory response could be mediated through the transcriptional and translational regulation of Nrf2 signaling [273]. Recent studies with Nrf2-deficient mice have shown that Nrf2 plays a role in protecting the liver from xenobiotic-initiated hepatocarcinogenesis [274].

Rajasekaran et al. have studied resveratrol’s ability to prevent or treat hepatocellular carcinoma by administering resveratrol, starting at the time of DENA injection or for 15 days after the development of hepatocellular carcinoma [269]. Resveratrol treatment at both time points also reduced cell crowding and alteration in the cellular architecture, and decreased the liver size compared with control rats treated with DENA [269]. In the DENA-induced hepatocellular carcinoma model, administration of resveratrol inhibited the formation of hepatocyte nodules via down-regulating Hsp70 and COX-2 expression, through lowering the translocation of NF-κB from the cytoplasm to the nucleus [275]. Another study using the same administered dose of resveratrol also determined that the levels and expressions of hepatic TNF-α, IL-1β, and IL-6 induced by DENA can be reversed [276]. Resveratrol also exhibited a remarkable anti-angiogenic effect during the development of DENA-induced hepatocellular carcinogenesis, perhaps by blocking VEGF expression via the down-regulation of HIF-1α [277].

Resveratrol considerably lowered the cell count of a swiftly growing tumor (Yoshida AH-130 ascites hepatoma) injected into rats, thereby triggering apoptosis and cell accumulation in the G2/M phase [228]. It was further demonstrated that the inhibition of cell cycle progression involved reductions in the expression of p34cdc2 and cyclin B1 in murine transplantable liver tumors after resveratrol administration [230]. It has also been reported that resveratrol had anti-tumor-growth and anti-metastasis effects in Donryu rats that had an ascites AH109A hepatoma cell line subcutaneously implanted [227].

In another study, resveratrol inhibited tumor growth and angiogenesis in a hepatoma xenograft mouse model [278]. Salado et al. used B16 melanoma (B16M) cells to study the effects of resveratrol treatment on hepatic metastasis caused mainly by the production of pro-inflammatory cytokines [279]. Lin et al. investigated the effects of treatment with resveratrol on the precancerous stage of liver carcinogenesis in spontaneously induced hepatocellular carcinoma in HBx transgenic mice [272]. Resveratrol supplementation significantly reduced the incidence of hepatocellular carcinoma and increased the latency of tumor formation. Resveratrol inhibited hepatic lipogenesis and intracellular ROS, and the results from liver cancer models have been consistently positive, indicating the potential benefit of resveratrol in hepatocellular carcinoma prevention and/or therapy.

## 12. Pancreatic Cancer

Several lines of evidence suggests that age, being overweight, pancreatitis and family history of pancreatic cancer are the major risk factor for the development of pancreatic cancer. Within a xenograft mouse model, resveratrol delayed or suppressed the promotion of pancreatic cancer via inhibiting the activity of leukotriene A4 hydrolase (LTA_4_H), which stimulates the generation of pro-inflammatory cytokines and mediators [280], and also stimulates cancer cell proliferation [281,282]. Resveratrol blocked the tumor development of PANC-1 cells orthotopically implanted in nude mice, with augmented expression of apoptosis/cell cycle arrest proteins including Bim, p27, and cleaved caspase-3, and reduced cell survival/proliferation markers including PCNA expression and the phosphorylation of PI3K, ERK, Akt, FOXO3a (Ser253), and p-FOXO1 (Ser256) in tumor tissues [283]. Resveratrol treatment inhibited the formation and development of pancreatic cancer in Kras^G12D^ transgenic mice that spontaneously develop pancreatic tumors [284]. However, dietary resveratrol had no anti-carcinogenic effect on BOP (*N*-nitrosobis(2-oxopropyl)amine)-induced pancreatic carcinogenesis in hamsters [285]. Further studies are necessary for additional preclinical evaluation of the efficacy of resveratrol in treating pancreatic cancer.

## 13. Lung Cancer

In preclinical models, lung carcinogenesis is known to be induced by a variety of agents, including diethylnitrosamine (DEN), nitrosamine 4-(methyl-nitrosamino)-1-(3-pyridyl)-1-butanone (NNK), uracil mustard, vinyl carbamate, urethane, MNU, and BP [11]. In the BP-induced mouse lung carcinogenesis model, resveratrol treatment lowered the level of BP diolepoxide (BPDE)-DNA adducts [286], improved the ultrahistoarchitecture [287], and reduced the size of tumor nodules by increasing pulmonary caspase-3 and -9 activity. It also abrogated glucose uptake/turnover, reduced the serum lactate dehydrogenase (LDH) activity (which is heightened in cancer cells), and lowered the p-p53 levels at Ser15 (the hyperphosphorylation of which can result in the inactivation of p53) [288]. In Lewis lung carcinoma cell xenograft models, treatment with resveratrol reduced the growth of tumors [218,221]. It has been also discovered that treatment with resveratrol reduced the development of A549 and MSTO-211H xenografts in mice [219,289,290].

Resveratrol’s anti-tumor effects in A549 xenografts were reduced in Forkhead box protein C2 (FOXC2)-overexpressing A549 xenografts, which suggests that resveratrol possibly induces anti-tumor activity through FOXC2 [289]. Another study discovered that resveratrol did not affect the development of Lewis lung carcinoma implanted in mice, but demonstrated an evident anti-metastatic effect, decreasing both the weight and number of lung metastases [220]. However, resveratrol used to supplement the diet did not affect lung tumor multiplicity in BP plus NNK-induced lung carcinogenesis in A/J mice [291]. Similarly, in BP-induced lung carcinogenesis, resveratrol did not cause a change in the expression levels of BP-metabolizing genes (such as CYP1A1 and CYP1B1) and the number of B[a]P-protein adducts in lung tissues [292]. Another study found that both the natural Egr-1 promoter and the synthetic promoter triggered the expression of GADD45α when used with resveratrol, and then suppressed the proliferation of A549 lung cancer cells and induced apoptosis [293].

## 14. Other Cancers

Resveratrol provides considerable protection against the induction of cancer within the oral cavity [294] and the esophagus [295], among other tissues. Its cancer chemopreventive activity aside, resveratrol can also inhibit the development and/or induce the regression of established tumors in xenograft models for cancers of the ovaries [296], urinary bladder [79], stomach [297], and head and neck [298,299]. Resveratrol treatment effectively suppressed the growth rate of and augmented apoptosis in neuroblastoma; this was accompanied by the up-regulation of cyclin E and the down-regulation of p21 [300]. It has recently been demonstrated that resveratrol considerably reduced tumor growth via inducing apoptosis, which involved direct activation of the mitochondrial intrinsic apoptotic pathway in the NGP and SK-N-AS xenograft models of human neuroblastoma [301]. Resveratrol caused significant inhibition of cerebral tumors through inducing apoptosis and inhibiting angiogenesis induced by glioma [302]. Rats that had undergone resveratrol treatment had lower growth rates of glioma, which correlated with the blood flow of tumors (signified by the color Doppler vascularity index) and density of microvessels.

Resveratrol’s anti-angiogenic effect has caused researchers to investigate if it could inhibit the development of a murine fibrosarcoma; water supplemented with resveratrol indeed significantly inhibited the development of T241fibrosarcoma in mice via suppressing angiogenesis [303]. Resveratrol’s in vivo anti-cancer effects were studied in N-nitrosomethyl-benzylamine (NMBA)-induced esophageal tumorigenesis in rats. Resveratrol suppressed both the size and number of NMBA-induced esophageal tumors per rat through targeting prostaglandin E2 and COXs [304]. In a gastric cancer xenograft nude mouse model, resveratrol inhibited the growth of tumors, with reductions in the expression of cyclin D1, Ki67, CDK4, and CDK6, and increases in the expression of p16, p21, and β-Gal [305]. Resveratrol considerably inhibited carcinoma development when it was injected in close proximity to the carcinoma in a tumor model created by transplanting human primary gastric cancer cells into the subcutaneous tissue of nude mice [297]. Resveratrol induced apoptosis in implanted tumor cells via down-regulation of the apoptosis-regulated gene Bcl-2 and up-regulation of the apoptosis-regulated gene Bax. For the anti-tumor effects in head and neck cancer, resveratrol suppressed tumor stemness via lowering the expression of mesenchymal-like protein (Vimentin) and stemness markers (Oct4 and Nestin), inducing epithelial protein expression (E-cadherin) [299], and increasing γ-histone 2AX (a DNA damage marker) and cleaved caspase-3 expression [298]. In an ovarian cancer model, resveratrol abrogated the development of NuTu-19 ovarian cancer cells in vitro. However, in vivo, when NuTu-19 cells were injected into the ovarian bursa of rats and the rats were fed with resveratrol (100 mg/kg) mixed in their diet for 28 days, the growth of the ovarian tumors was not significantly inhibited [306].

## 15. Clinical Trials with Resveratrol

Although it is clear that resveratrol has shown excellent anti-cancer properties, most of the studies were performed in cell-culture and pre-clinical models. These physiological effects of resveratrol were also investigated in humans because it cannot be assumed that the results of tests in animal models will hold true for humans, because of differences in genetics and metabolism profile. The pharmacokinetics, metabolism, and toxicity of resveratrol have been assessed in healthy volunteers and cancer patients [307,308,309]. Resveratrol is metabolized swiftly, mainly into glucuronide and sulfate conjugates that are excreted via the urine. Because of the poor bioavailability of resveratrol due to its extensive metabolism, large doses (up to a maximum of 5 g/day) have been utilized by researchers. These studies have shown that resveratrol seems to be well tolerated and safe. However, adverse effects including diarrhea, nausea, and abdominal pain were observed in subjects taking more than 1 g of resveratrol daily [307]. Subsequent clinical trials are currently investigating this dose limit [307,310]. Resveratrol’s poor bioavailability is a significant issue with regard to extrapolating its effects to humans, and various approaches have been created to enhance its bioavailability [311], including consuming it with various foods [312], using it in combination with an additional phytochemical piperine [313], and using a prodrug approach [314], micronized powders [315,316], or nanotechnological formulations [317,318,319].

The effect of resveratrol in cancer patients has been investigated in a few clinical trials (Table 2). The first clinical trial dealing with resveratrol and cancer was performed by Nguyen et al. [320]. They examined the effects of freeze-dried grape powder (GP) (containing resveratrol and resveratrol derived from plants) on the Wnt signaling pathway, which is known to be involved in colon carcinogenesis [321], in regular colon cancer and colonic mucosa. GP administration (80 g/day containing 0.07 mg of resveratrol) for two weeks resulted in decreased Wnt target gene expression within regular mucosa, but had no effect on cancerous mucosa. This indicates that GP or resveratrol may play a beneficial part in the prevention of colon cancer, rather than in the treatment of established colon cancer. Patel et al. studied the effects of the administration of resveratrol at 0.5 or 1 g/day for eight days on proliferation marker Ki-67 expression in colorectal tissue, and reported a 5% decrease in the proliferation of tumor cells [322]. In colorectal cancer patients with hepatic metastasis, SRT501 (a micronized resveratrol formulation manufactured by Sirtris Pharmaceuticals, a GSK Company, Cambridge, MA, USA) supplementation at 5 g/day for two weeks increased the amount of cleaved caspase-3 within hepatic tissue, which suggests that there was increased apoptosis of cancerous tissue compared with subjects treated with a placebo [315].

In a muscadine grape skin extract phase 1 study with biochemically recurrent prostate cancer patients who were assigned to a high dose (4000 mg/patient) of pulverized muscadine grape (*Vitis* *rotundifolia*) skin that contains ellagic acid, quercetin, and resveratrol was found to be safe and warrants further investigation in dose-evaluating phase II trial [323]. In another randomized placebo controlled clinical study using two doses of resveratrol (150 mg or 1000 mg resveratrol daily) for 4 months was found to significantly lowered serum levels of androstenedione, dehydroepiandrosterone and dehydroepiandrosterone-sulphate, whereas prostate size was unaffected in benign prostate hyperplasia patients [324].

Primary protein carbonylation has been found to be increased several folds in presence of high levels of reactive oxygen species (ROS) such as superoxide anion free radical (O_2_^−^) and nitric oxide free radical (NO) and other reactive free radicals, such as hydrogen peroxide (H_2_O_2_), hydroxyl radical (HO), and peroxynitrite anion (ONOO^−^). There are several sources of ROS in the digestive tract and several microbes present in the colon produce a large amount of ROS inside the cells are by products of mitochondrial respiration in aerobic metabolism, and in chronic inflammation, a large amount of ROS is produced by neutrophil phagocytosis of bacteria, granular materials, or soluble irritants [325,326]. The oxidative decomposition of polyunsaturated fatty acids can initiate chain reactions that lead to the formation of a variety of carbonyl species (three to nine carbons in length), the most reactive and cytotoxic being α,β-unsaturated aldehydes also referred to as electrophilic carbonyls. These include acrolein, glyoxal, methylglyoxal, crotonaldehyde, malondialdehyde, and 4-hydroxynonenal. Reactive ketones or aldehydes that can be reacted by 2,4-dinitrophenylhydrazine (DNPH) to form 2,4-dinitrophenylhydrazone (DNP). Ulcerative colitis (UC) is a type of chronic inflammatory bowel disease (IBD) in which oxidative stress plays a critical role in its pathogenesis and malignant progression to colorectal cancer (CRC) [327,328]. Oxidative activation of transcription factors NF-*κ*B stimulates expression of a variety of pro-inflammatory cytokines in the intestinal epithelial cells, such as TNF-*α*, IL-1, IL-8, and COX-2, and promotes inflammation and carcinogenesis. Oxidative stress also activates mitogen-activated protein (MAP) kinase (MAPK) signaling pathways. The human gastrointestinal tract is exposed to carbonyl threats such as consumption red meat, alcoholic beverages and smoking increases protein carbonylation, inflammation and initiation of tumor development. However, dietary intake of green leafy vegetables, fruits, fish and wine has shown to decrease protein carbonylation [329]. It has also been reported that resveratrol supplementation at 5 mg/day for six days increased the degree of protein carbonyl concentrations and cytoprotective enzyme NQO1 in colorectal mucosa tissues from patients with colorectal cancer, compared with their control subjects [330]. However, contrary to these positive findings, some evidence that resveratrol supplementation may have adverse effects in certain cancer patients also exist. In a phase II clinical trial involving multiple myeloma patients, SRT501 supplementation at 5 g/day caused several unexpected adverse effects, including nephrotoxicity, which may have led to the death of one patient [316]. However, this high dose of SRT501 was determined to be safe in other clinical trials involving several healthy and diseased populations [315,316]. There are very low amounts of human data regarding the efficacy of resveratrol in cancer treatment. Since most of these clinical trials have had a small patient sample size and used different doses and different routes of resveratrol, the data from human clinical studies have shown inconsistent outcomes of resveratrol administration.

In addition to the effects in subjects with cancer, the effect of resveratrol in subjects with a higher cancer risk has also been demonstrated. For instance, resveratrol supplementation at 50 mg two times per day for 12 weeks reduced the DNA methylation of the tumor-suppressor gene *Ras* association domain-containing protein 1 (RASSF1A) in the breasts of women with higher risk of breast cancer [331]. It has also been shown that resveratrol supplementation at 1 g/day for 12 weeks increases the concentrations of sex steroid hormone binding globulin (SHBG), which has been linked to a reduction in the risk of breast cancer [332], and has favorable effects on estrogen metabolism; thus, it can lower risk factors for breast cancer in obese and overweight postmenopausal women [333]. Another clinical study concentrated on resveratrol’s effects on potential biomarkers for cancer risk reduction. Circulating concentrations of insulin-like growth factor (IGF-1) and IGF-binding protein 3 (IGFBP-3) are linked to a higher risk of common cancers [334]. Brown et al. showed that resveratrol administration at 2.5 g/day for 29 days resulted in a reduction of the circulating levels of IGF-1 and IGFBP-3 in healthy volunteers [335]. Their research suggests that resveratrol’s ability to decrease circulating IGF-1 and IGFBP-3 in humans may constitute an anti-carcinogenic mechanism. In another study, Chow et al. found that resveratrol administration at 1 g/day for four weeks modulated phase I isoenzymes (cytochrome P450) and phase II detoxification enzymes involved in carcinogen activation and detoxification [310]. However, these beneficial effects are mostly minimal and sometimes controversial. Nevertheless, it seems that resveratrol has had some beneficial effects with regard to the prevention and treatment of cancer. Therefore, the efficacy and safety of resveratrol in human trials must be further investigated to better understand and develop its therapeutic potential for cancer patients.

## 16. Conclusions and Future Perspectives

**Figure 2:**
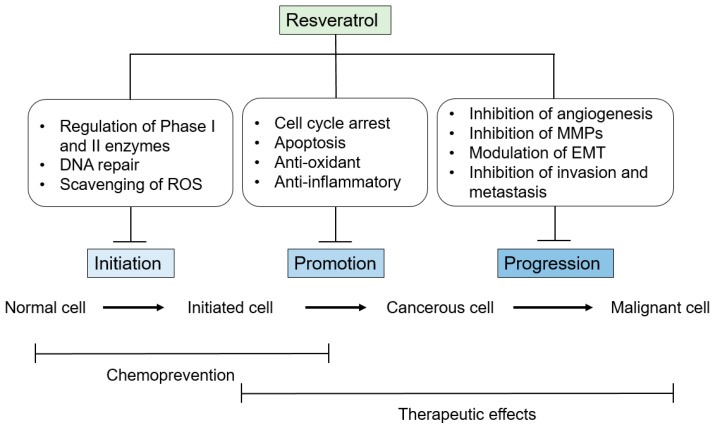
A schematic diagram summarizing the potential mechanism(s) underlying the anticancer effects of resveratrol.

Using a variety of in vivo and in vitro models, it has been proven that resveratrol is capable of attenuating the various stages of carcinogenesis, some of which are briefly described in Figure 2. A vast body of experimental in vivo and in vitro studies and a few clinical trials has presented evidence of resveratrol’s great potential as an anti-cancer agent, both for the prevention and therapy of a large range of cancers. Resveratrol has a very low toxicity, and, although it has multiple molecular targets, it acts on different protective and common pathways that are usually altered in a great number of tumors. This suggests that resveratrol may be more suitable for use as an anti-carcinogen and it can also effectively exert it antineoplastic effects in conjunction with diverse chemotherapeutics and targeted therapies. The ability to prevent carcinogenesis includes the inhibition of oxidative stress, inflammation, and cancer-cell proliferation, and the activation of tightly regulated cell-death mechanisms. Due to the complexity and number of cellular processes involved, however, more studies must be performed to completely understand how resveratrol could be used to prevent the development of cancer. Moreover, resveratrol’s poor bioavailability in humans has been a critical concern with regard to the translation of basic research findings to the development of therapeutic agents. Although human clinical trials have produced positive findings, many conflicting results remain, which may be partly because of the dosing protocols employed. To augment resveratrol’s bioavailability and as a potential adjuvant, active research should be focused on resveratrol delivery systems, formulations, and modulation of resveratrol metabolism, and resveratrol’s possible interactions with other compounds, as well as the development of more bioavailable analogs of the compound.

## Figures and Tables

**Table 1:** In vivo anti-cancer effects of resveratrol.

Cancer Model	Animal Model	Dose	Outcome	References
Skin	DMBA/TPA model in female CD-1 mice	1, 5, 10, 25 μmol topically twice/week for 18 weeks	Incidence↓ Number of tumors per mouse↓	[46]
	Mouse xenograft models of A431 cells	10, 20, 40 μg i.p. for 14 days	Xenograft volume↓ Free radical scavenging Incidence↓ Number of tumors per mouse↓	[198]
	DMBA-initiated and TPA-promoted papillomas in female ICR mice	85 nmol/L for 21 days; topical application	Prevent onset of skin tumor	[199]
	DMBA/TPA model in CD-1 mice	1, 5, 10, 25 μmol Twice/week, for 18 Wk; topical application	Skin tumor incidence↓ Apoptosis↑; p53↑; Bax↑; cytochrome C↑; APAF↑; Bcl2↓	[200]
	DMBA-TPA–model in male Swiss albino mice	50 μmol/mouse for 3–24 week; topical application	Inhibits photocarcinogenesis; Cox2↓; lipid peroxidation↓; ODC↓	[201]
	UVB-mediated photocarcinogenesis in female SKH-1 mice	25 μmol/mouse; topical application	Decrease hyperplasia; p53↑; Cox2↓; ODC↓; survivin↓ mRNA and protein	[202]
	UVB-induced skin hyperplasia in female SKH-1 mice	10 μmol/mouse; 7 times, on alternate days; topical application	Skin tumor incidence↓ ↑Survivin mRNA and protein; ↑ phospho-survivin; ↓Smac/DIABLO	[203,204].
	UVB-induced skin tumorigenesis in female SKH-1 mice	25, 50 μmol/mouse; twice/week for 28 weeks; topical application	Suppresses melanoma tumor growth	[205]
	C57Bl/6N mice transplanted with B16-BL6 melanoma cells	50 mg/kg b.w.; i.p. for 19 days		[206]
Breast	Spontaneous mammary tumor in female FVB/N HER-2/neu mice	4 μg/mouse/day in drinking water for 2 months	Onset of tumorigenesis↓ Tumor volume↓ Multiplicity↓ Apoptosis↑	[207]
	Female athymic mice xenograft models of MDA-MB-231 cells	25 mg/kg/day i.p. daily for 3 weeks	Tumor volume↓ TUNEL staining↓ Microvessel density↓	[208]
	Female Balb/c mice xenograft with cigarette smoke condensate-transformed, MCF-10A-Tr cells in mammary fat pad	40 mg/kg/day orally for 30 days	Tumor volume↓	[209]
	DMBA-induced mammary carcinogenesis in female Sprague-Dawley rats	10 ppm mixed in diet; for 127 days	Suppressed tumor growth NF-κB↓;Cox2↓; MMP9↓	[210]
	DMBA-induced mammary carcinogenesis in female Sprague-Dawley rats	100 mg/kg b.w. mixed in diet; for 25 weeks	Suppressed tumor growth Cell proliferation↓ Apoptosis↑	[211]
	MNU-induced mammary tumorigenesis in female Sprague-Dawley rats	100 mg/kg b.w. by oral gavage for 127 days	Estrogen modulation Reduces tumor growth	[212]
	MDA-MB-231 breast tumor xenograft model	25 mg/kg b.w, by i.p., for 3 weeks	Inhibits tumor growth Apoptosis↑ Angiogenesis↓	[208]
	Female HER-2/neu transgenic mice model	0.2 mg/kg b.w in drinking water for 2 months	Delays the development and reduces the metastatic growth of spontaneous mammary tumors Apoptosis↑ ↓HER-2/neu mRNA and protein	[207]
	MDA-MB-231 breast tumor xenograft model in female athymic nu/nu mice	5 and 25 mg/kg b.w., thrice a week by oral gavage for 117 days,	In combination with quercetin and catechin retards the growth of tumor	[213]
Prostate	Athymic nude mice xenograft models of PC-3 cells	30 mg/kg/day Thrice/week, total 6 weeks	Tumor volume↓ Cell proliferation↓ Apoptosis↑ Number of blood vessels↓	[214]
	Male nude mice xenograft models with Du145-EV-Luc or Du145-MTA1 shRNA-Luc in anterior prostate	50 mg/kg/day i.p. daily 14 days after implantation, total 6 weeks	Tumor growth↓ Progression, local invasion↓ Spontaneous metastasis↓ Angiogenesis↓ Apoptosis↑	[215]
	Transgenic adenocarcinoma of mouse prostate (TRAMP) model	625 mg/kg mixed in diet for 7–23 weeks	ER-β ↑; IGF-I ↑; ↓phospho-ERK-1;↓ERK-2	[216]
	Transgenic rat adenocarcinoma of prostate (TRAP) model	50, 100 or 200 μg/ml in drinking water for 7 weeks	Apoptosis ↑; ↓AR; ↓GK11 mRNA	[217]
Lung	Female C57BL/6 mice xenograft models of LLC tumors	0.6, 2.5 or 10 mg/kg/day i.p. daily for 21 days	Tumor volume/weight↓ Metastasis to lung↓	[218]
	Nude mice xenograft models of A549	15, 30 or 60 mg/kg i.v. daily for 15 days	Tumor volume↓	[219]
	C57BL/6 mice implanted with Lewis lung carcinoma lung tumor model	5 and 25 mg/kg, i.p. for 15 days	Metastasis↓ Angiogenesis↓	[220]
	C57BL/6 mice implanted with Lewis lung carcinoma lung tumor model	20 mg/kg, i.p. for 17 days	Angiogenesis↓ Apoptosis ↑	[221]
Colon	DMH models in male Wistar rats	8 mg/kg/day orally daily for 30 weeks	Incidence↓, Tumor volume↓, Tumor burden/rat↓ Histopathological lesions DMH↓	[222]
	BP models in male Apc^Min^ mice	45 μg/kg/day orally, for 60 days	Number of colon adenomas↓ Dysplasia occurrence↓	[223]
	AOM induced colon cancer in male F344 rats	200 μg/kg b.w. in drinking water	Bax↑; p21↑	[224]
	ApcMin/+ mice model	0.01% in drinking water for 7 weeks	Reduce formation of tumor in small intestine cyclin D1 and D2↓	[225]
	ApcMin/+ mice model	240 mg/kg b.w. mixed in diet for 10–14 weeks	Suppress intestinal adenoma formation Cox1 and 2↓; PGE2↓	[226]
Liver	Male Donryu rats xenograft models of AH109A cells	10, 50 ppm in diet for 20 days	Tumor weight↓ Metastasis↓	[227]
	Male Wistar rats implanted with AH- 130 hepatoma cells	1 mg/kg; 7 days; i.p.	Tumor weight↓ Apoptosis↑ ↑cells at G2/M	[228]
	BALB/c mice implanted with H22 hepatoma cells	500, 1000, 1500 mg/kg; 10 days; abdominal injection	Immunomodulatory activity↑	[229]
	BALB/c mice implanted with H22 hepatoma cells	5, 10, 15 mg/kg; 10 days; abdominal injection	Tumor volume↓ Apoptosis↑ cyclin B1↓; p34cdc2↓	[230]
	BALB/c mice implanted with H22 hepatoma cells	5, 10, 15 mg/kg; 10 days; abdominal injection	Synergistic anti-tumor effect in combination with 5-FU; S-phase arrest	[231]
	Female BALB/c mice implanted with HepG2 cells	15 mg/kg; every alternate day for 21 days; i.p.	Tumor growth↓ Apoptosis↑ Caspase 3↑	[232]
	DENA-initiated GST-P-positive hepatic pre-neoplastic foci in male Sprague–Dawley rats	15% (*w*/*w*) grape extract in diet; 11 weeks	Tumor growth↓ Lipid peroxidation↓ Fas ↓	[233]
	DENA-initiated and PB-promoted hepatocyte nodule formation in female Sprague–Dawley rats	50, 100, 300 mg/kg; 20 weeks; diet	Tumor growth↓ Apoptosis↑ Cell proliferation↓ Bcl2↓; Bax↑	[234]

↓: downregulated; ↑: upregulated; UVB: ultraviolet B; DMBA: 7,12-Dimethylbenz[a]anthracene; MNU: methyl-N-nitrosourea; AOM: azoxymethane; DENA: diethylnitrosamine; GST-P: glutathione S-transferase; PB: phenobarbital ; p53: tumor protein p53; Bax: Bcl-2-associated-X-protein; APAF: Apoptotic protease activating factor 1; Bcl2: B-cell lymphoma 2; Cox: cyclooxygenase; ODC: ornithine decarboxylase; Smac/DIABLO: Second mitochondriaderived activator of caspases /Diablo homolog; TUNEL: Terminal deoxynucleotidyl transferase dUTP nick end labeling; NF-κB: nuclear factor kappa-light-chain-enhancer of activated B cells; MMP9: matrix metalloproteinase nine; HER-2: human epidermal growth factor receptor 2; ER-β: estrogen receptor beta; IGF-I: insulin-like growth factor 1ERK: extracellular regulated kinase; AR: androgen receptor; GK11: glandular kallikrein 11; DMH: 1,2-dimethylhydrazine ; PGE2: prostaglandin E2; 5-FU: 5-fluorouracil.

**Table 2:** Selected clinical trials evaluating the effect of resveratrol in cancer patients.

Participants	Resveratrol Formulation and Dosages	Outcome	References
Colorectal cancer patients (*n* = 8)	Grape powder (80 or 120 g/day) or Resveratrol (20 or 80 mg/day) for 2 weeks	Inhibition of Wnt target gene expression in normal colonic mucosa.	[320]
Colorectal cancer patients (*n* = 20)	Resveratrol (0.5 or 1g) for 8 days	Reduction of Ki-67 levels by 5 and 7% in cancerous and normal tissue, respectively.	[322]
Colorectal cancer patients with hepatic metastasis (*n* = 6)	Micronized resveratrol (SRT5001, 5 g) for 14 days	Detection of resveratrol in hepatic tissue and increased (39%) content of cleaved caspase-3 in malignant hepatic tissue.	[315]
Multiple myeloma patients (*n* = 24)	Micronized resveratrol (SRT5001, 5 g) for 20 days in a 21 day cycle up to 12 cycles	Unacceptable safety profile and minimal efficacy in patients with relapsed/refractory multiple myeloma highlighting the risks of novel drug development in such populations.	[316]
Biochemically recurrent prostate cancer patients (*n* = 14)	Pulverized muscadine grape skin extract (MPX) 4000 mg/patient	MPX was found to be safe and warrants further investigation in dose-evaluating phase II trial	[323]
Benign prostate hyperplasis patients (*n* = 66)	Two doses of resveratrol (150 mg or 1000 mg resveratrol daily) for 4 months	Significantly lowered the serum levels of androgens with no changes in prostate tumor growth.	[324]
